# An alternative extragradient projection method for quasi-equilibrium problems

**DOI:** 10.1186/s13660-018-1619-9

**Published:** 2018-01-30

**Authors:** Haibin Chen, Yiju Wang, Yi Xu

**Affiliations:** 1 School of Management Science, Qufu Normal University, Rizhao Shandong, China; 2 Department of Applied Mathematics, Southeast University, Nanjing, China

**Keywords:** 90C30, 15A06, Quasi-equilibrium problems, Extragradient projection method, Pseudomonotonicity, Multi-valued mapping

## Abstract

For the quasi-equilibrium problem where the players’ costs and their strategies both depend on the rival’s decisions, an alternative extragradient projection method for solving it is designed. Different from the classical extragradient projection method whose generated sequence has the contraction property with respect to the solution set, the newly designed method possesses an expansion property with respect to a given initial point. The global convergence of the method is established under the assumptions of pseudomonotonicity of the equilibrium function and of continuity of the underlying multi-valued mapping. Furthermore, we show that the generated sequence converges to the nearest point in the solution set to the initial point. Numerical experiments show the efficiency of the method.

## Introduction

The equilibrium problem has been considered as an important and general framework for describing various problems arising in different areas of mathematics, including optimization problems, mathematical economic problems and Nash equilibrium problems. As far as we know, this formulation has been followed for a long time by several studies on equilibrium problems considered under different headings such as quasi-equilibrium problem, mixed equilibrium problem, ordered equilibrium problem, vector equilibrium problem and so on [1–4]. It should be noted that one of the interests of this common formulation is that many techniques developed for a particular case may be extended, with suitable adaptations, to the equilibrium problem, and then they can be applied to other particular cases [5–16]. In this paper, we mainly deal with existence of solutions and approximate solutions of the quasi-equilibrium problem.

Let $X\subset\mathbb{R}^{n}$ be a nonempty closed convex set, *K* be a point-to-set mapping from *X* onto itself such that, for any $x\in X$, $K(x)$ is a nonempty closed convex set of *X*, and let $f:X\times X \to R$ be a function such that, for any $x\in X$, $f(x,x)=0$ and $f(x,\cdot )$ is convex on *X*. The quasi-equilibrium problem $\operatorname{QEP}(K,f)$ is to find a vector $x^{*}\in K(x^{*})$ such that

1.1 $$ f\bigl(x^{*},y\bigr)\ge0,\quad\forall y\in K\bigl(x^{*}\bigr). $$

Throughout this paper, we denote the solution set by $K^{*}$.

Certainly, when $f(x,y)=\langle F(x),y-x\rangle$ with *F* being a vector-valued mapping from *X* to $\mathbb{R}^{n}$, then the quasi-equilibrium problem reduces to the generalized variational inequality or quasi-variational inequality problem [17–20] which is to find vector $x^{*}\in K(x^{*})$ such that

1.2 $$ \bigl\langle F\bigl(x^{*}\bigr),y-x^{*}\bigr\rangle \ge0, \quad \forall y \in K\bigl(x^{*}\bigr). $$

To move on, we recall the classical equilibrium problem, and classical Nash equilibrium problem (NEP) [21]. Assume the function $f_{i}:\mathbb{R}^{n}\to R$ is continuous, and suppose $K_{i}$ is a nonempty closed set in $R^{n_{i}}$ for $i=1,2,\ldots, N$ with $n=\sum_{i=1}^{N}n_{i}$. Suppose that there are *N* players such that each player controls the variables $x_{i}\in R^{n_{i}}$. Denote $x=(x_{1},\ldots, x_{N})$, and $x_{-i}=(x_{1},\ldots, x_{i-1}, x_{i+1},\ldots, x_{N})$. Player *i* needs to take an $x_{i}\in K_{i}\subset R^{n_{i}}$ that solves the following optimization problem:

$$\min_{x_{i}\in K_{i}}f_{i}(x_{i}, x_{-i}) $$

based on the other players’ strategies $x_{-i}$. If these *N* players do not cooperate, then each players’ strategy set may vary with other players’ strategies, that is, the *i*th player’s strategy set varies with the other players’ strategies $x_{-i}$. In this case, we need to use $K_{i}(x_{-i})$ instead of $K_{i}$ to indicate the *i*th player’s strategy set, and the *i*th player needs to choose a strategy $x^{*}_{i} \in K_{i}(x_{-i})$ that solves the following optimization problem:

$$\min_{x_{i}\in K_{i}(x_{-i})} f_{i}(x_{i}, x_{-i}). $$

In [22], the non-cooperative game model is called generalized Nash equilibrium problem (GNEP), which can be formulated as the quasi-equilibrium problem where the involved functions are nondifferentiable [23].

For the problem GNEP, when the functions $f_{i}(\cdot, x_{-i})$ are convex and differentiable, then the problem can be equivalently formulated as the quasi-variational inequalities (1.2) by setting

$$F(x)=\bigl(\nabla_{x_{i}}f_{i}(x)\bigr)_{i=1}^{N} $$

and $K(x)=\prod_{i=1}^{N}K_{i}(x_{-i})$. When the function $f_{i}(\cdot, x_{-i})$ is convex and nondifferentiable, then the GNEP reduces to the quasi-equilibrium problem (1.1) [24] via the Nikaido Isoda funtion

$$f(x,y)=\sum_{i=1}^{N}\bigl[f_{i}(y_{i},x_{-i})-f_{i}(x_{i},x_{-i}) \bigr]. $$

On the other hand, the quasi-equilibrium problem (QEP) has received much attention of researchers in mathematics, economics, engineering, operations research, etc. [17, 22]; for more information see [19, 25, 26]. There are many solution methods for solving QEP. Recently, [27] considered an optimization reformulation approach with the regularized gap function. Different from the variational inequalities problem, the regularized gap function is in general not differentiable, but only directional differentiable. Furthermore, supplementary conditions must be imposed to guarantee that any stationary point of these functions is a global minimum, since the gap functions is nonconvex [28]. It should be noted that such conditions are known for variational inequality problem but not for QEP. So, [23] proposed several projection and extragradient methods rather than methods based on gap functions, which generalized the double-projection methods for variational inequality problem to equilibrium problems with a moving constraint set $K(x)$.

It is well known that the extragradient projection method is an efficient solution method for variational inequalities due to its low memory and low cost of computing [29, 30]. Based on those advantages, it was extended to solve QEP recently [20, 23, 31] and this opened a new approach for solving the problem. An important feature of this method is that it has the contraction property in the sense that the generated sequence has contraction property with respect to the solution set of the problem [29], *i.e.*

$$\bigl\Vert x^{k+1}-x^{*} \bigr\Vert \leq \bigl\Vert x^{k}-x^{*} \bigr\Vert ,\quad\forall k\geq0, x^{*}\in K^{*}. $$

The numerical experiments given in [20, 23, 31] show that the extragradient projection method is a practical solution method for the QEP.

It should be noted that not all the extragradient projection methods have the contraction property [32]. In that case, it may not slow down the convergence rate significantly. Although the extragradient projection method has no contraction property, it still has a good numerical performance [32]. Now, a question can be posed naturally, can this method be applied to solve the QEP? And if so, how about its performance? This constitutes the main motivation of this paper.

Inspired by the work [23, 32], we propose a new type of extragradient projection method for the QEP in this paper. Different from the extragradient projection method proposed in [23], the generated sequence by the newly designed method possesses an expansion property with respect to the initial point, *i.e.*,

$$\bigl\Vert x^{k+1}-x^{k} \bigr\Vert ^{2}+ \bigl\Vert x^{k}-x^{0} \bigr\Vert ^{2}\leq \bigl\Vert x^{k+1}-x^{0} \bigr\Vert ^{2}. $$

The existence results for (1.1) are established under pseudomonotonicity condition of the equilibrium function and the continuity of the underlying multi-valued mapping. Furthermore, we show that the generated sequence converges to the nearest point in the solution set to the initial point. Numerical experiments show the efficiency of the method.

The remainder of this paper is organized as follows. In Section 2, we recall some concepts and related conclusions to be used in the sequel. The newly designed method and its global convergence are developed in Section 3. Some preliminary computational results and experiments are presented in Section 4.

## Preliminaries

Let *X* be a nonempty closed convex set in $\mathbb{R}^{n}$. For any $x\in\mathbb{R}^{n}$, the orthogonal projection of *x* onto *X* is defined as

$$y_{0}=\arg\min\bigl\{ \Vert y-x \Vert \mid y\in X \bigr\} , $$

and denote $P_{X}(x)=y_{0}$. A basic property of the projection operator is as follows [33].

### Lemma 2.1

*Suppose* *X* *is a nonempty*, *closed and convex subset in* $\mathbb{R}^{n}$. *For any* $x, y\in \mathbb{R}^{n}$, *and* $z\in X$, *we have*

(i) $\langle P_{X}(x)-x, z-P_{X}(x) \rangle\geq0$;
(ii) $\Vert P_{X}(x)-P_{X}(y) \Vert^{2} \leq \Vert x-y \Vert^{2}- \Vert P_{X}(x)-x+y-P_{X}(y) \Vert^{2}$.

### Remark 2.1

The first statement in Lemma 2.1 also provides a sufficient condition for vector $y\in X$ to be the projection of vector *x*, *i.e.*, $y=P_{X}(x)$ if and only if

$$\langle y-x, z-y \rangle\geq0,\quad \forall z\in X. $$

To proceed, we present the following definitions [34].

### Definition 2.1

Suppose *X* is a nonempty subset of $\mathbb{R}^{n}$. The bifunction $f:X\times X \rightarrow R$ is said to be

(i) strongly monotone on *X* with $\beta> 0$ iff
  $$f(x,y)+f(y,x)\leq-\beta \Vert x-y \Vert ^{2},\quad\forall x, y \in X; $$
(ii) monotone on *X* iff
  $$f(x,y)+f(y,x)\leq0, \quad\forall x,y \in X; $$
(iii) pseudomonotone on *X* iff
  $$f(x,y)\geq0\quad \Rightarrow\quad f(y,x)\leq0,\quad\forall x,y \in X. $$

### Definition 2.2

Suppose *X* is a nonempty, closed and convex subset of $\mathbb{R}^{n}$. A multi-valued mapping $K: X \to2^{ \mathbb{R}^{n}}$ is said to be

(i) upper semicontinuous at $x\in X$ if for any convergent sequence $\{ x_{k}\}\subset X$ with *x̄* being the limit, and for any convergent sequence $\{y_{k}\}$ with $y_{k}\in K(x_{k})$ and *ȳ* being the limit, then $\bar{y}\in K(\bar{x})$;
(ii) lower semicontinuous at $x\in X$ if given any sequence $\{x^{k}\}$ converging to *x* and any $y\in K(x)$, there exists a sequence $\{y^{k}\} $ with $y^{k}\in K(x^{k})$ converges to *y*;
(iii) continuous at $x\in X$ if it is both upper semicontinuous and lower semicontinuous at *x*.

To end this section, we make the following blanket assumption on bifunction $f:X\times X\to R$ and multi-valued mapping $K: X \to2^{ \mathbb{R}^{n}}$ [20, 23].

### Assumption 2.1

For the closed convex set $X\subset \mathbb{R}^{n}$, the bifunction *f* and multi-valued mapping *K* satisfy:

(i) $f(x,\cdot)$ is convex for any fixed $x\in X$, *f* is continuous on $X\times X$ and $f(x,x)=0$ for all $x\in X$;
(ii) *K* is continuous on *X* and $K(x)$ is a nonempty closed convex subset of *X* for all $x\in X$;
(iii) $x\in K(x)$ for all $x \in X$;
(iv) $S^{*}=\{x\in S \mid f(x,y)\geq0, \forall y \in T \}$ is nonempty for $S=\bigcap_{x\in X}K(x)$ and $T=\bigcup_{x\in X}K(x)$;
(v) *f* is pseudomonotone on *X* with respect to $S^{*}$ *i.e.* $f(x^{*}, y)\geq0\Rightarrow f(y,x^{*})\leq0$, $\forall x^{*}\in S^{*}$, $\forall y\in X$.

As noted in [23], the assumption (iv) in Assumption 2.1 guarantees that the solution set of problem (1.1) $K^{*}$ is nonempty.

## Algorithm and convergence

In this section, we mainly develop a new type of extragradient projection method for solving QEP. The basic idea of the algorithm is as follows. At each step of the algorithm, we obtain a solution $y^{k}$ by solving a convex subproblem. If $x^{k}=y^{k}$, then stop with $x^{k}$ being a solution of the QEP; otherwise, find a trial point $z^{k}$ by a back-tracking search at $x^{k}$ along the direction $x^{k}-y^{k}$, and the new iterate is obtained by projecting $x^{0}$ onto the intersection of $K(x^{k})$ of two halfspaces which are, respectively, associated with $z^{k}$ and $x^{k}$. Repeat the process until $x^{k}=y^{k}$. The detailed description of our designed algorithm is as follows.

### Algorithm 3.1

**Step 0.**: Choose $c, \gamma\in(0, 1)$, $x^{0}\in X$, $k=0$.
**Step 1.**: For current iterate $x^{k}$, compute $y_{k}$ by solving the following optimization problem:
  $$\min_{y\in K(x^{k})} \biggl\{ f\bigl(x^{k},y\bigr)+ \frac{1}{2} \bigl\Vert y-x^{k} \bigr\Vert ^{2}\biggr\} . $$
  If $x^{k}=y^{k}$, then stop. Otherwise, let $z^{k}=(1-\eta_{k})x^{k}+\eta_{k} y^{k}$, where $\eta _{k}=\gamma^{m_{k}}$ with $m_{k}$ being the smallest nonnegative integer such that
  3.1 $$ f\bigl(\bigl(1-\gamma^{m}\bigr)x^{k}+ \gamma^{m} y^{k}, x^{k}\bigr)-f\bigl(\bigl(1- \gamma^{m}\bigr)x^{k}+\gamma^{m} y^{k}, y^{k}\bigr)\geq c \bigl\Vert x^{k}-y^{k} \bigr\Vert ^{2}. $$
**Step 2.**: Compute $x^{k+1}=P_{H_{k}^{1}\cap H_{k}^{2} \cap X}(x^{0})$ where
  $$\begin{gathered} H_{k}^{1}=\bigl\{ x\in \mathbb{R}^{n} \mid f\bigl(z^{k},x\bigr)\leq0 \bigr\} , \\ H_{k}^{2}=\bigl\{ x\in \mathbb{R}^{n} \mid \bigl\langle x-x^{k}, x^{0}-x^{k} \bigr\rangle \leq 0 \bigr\} . \end{gathered} $$
  Set $k=k+1$ and go to Step 1.

Indeed, for every $x^{k}\in K(x^{k})$, since $y^{k}\in K(x^{k})$, $z^{k}\in K(x^{k})$, so we have $K(x^{k})\cap H_{k}^{1}\neq\emptyset$ and $K(x^{k})\cap H_{k}^{2}\neq\emptyset$. To establish the convergence of the algorithm, we first discuss the relationship of the halfspace $H_{k}^{1}$ with $x^{k}$ and the solution set $K^{*}$.

### Lemma 3.1

*If* $x^{k}\neq y^{k}$, *then the halfspace* $H_{k}^{1}$ *in Algorithm *3.1 *separates the point* $x^{k}$ *from the set* $K^{*}$ *under Assumption *2.1. *Moreover*,

$$K^{*}\subseteq H_{k}^{1}\cap X, \quad \forall k\geq0. $$

### Proof

First, by the fact that $f(x,\cdot)$ is convex and

$$z^{k}=(1-\eta_{k})x^{k}+\eta_{k} y^{k}, $$

we obtain

$$0=f\bigl(z^{k},z^{k}\bigr)\leq(1-\eta_{k})f \bigl(z^{k}, x^{k}\bigr)+ \eta_{k} f \bigl(z^{k},y^{k}\bigr), $$

which can be written as

$$-f\bigl(z^{k},y^{k}\bigr)\leq\biggl(\frac{1}{\eta_{k}}-1 \biggr)f\bigl(z^{k},x^{k}\bigr). $$

By (3.1), we have

$$f\bigl(z^{k},x^{k}\bigr)\geq c\eta_{k} \bigl\Vert x^{k}-y^{k} \bigr\Vert ^{2}>0, $$

which means $x^{k} \notin H_{k}^{1}$.

On the other hand, by Assumption 2.1, it follows that $K^{*}$ is nonempty. For any $x\in K^{*}$, from the definition of $K^{*}$ and the pseudomonotone property of *f*, one has

$$f\bigl(z^{k},x\bigr)\leq0, $$

which implies that the curve $\partial H_{k}^{1}$ separates the point $x^{k}$ from the set $X^{*}$. Furthermore, by the definition of $K^{*}$, it is easy to see that

$$K^{*}\subseteq H_{k}^{1}\cap X, \quad \forall k\geq0, $$

and the desired result follows. □

The justification of the termination criterion can be seen from Proposition 2 in [23], and the feasibility of the stepsize rule (3.1), *i.e.*, the existence of point $m_{k}$ can be guaranteed from Proposition 7 in [23].

Next, to show that the algorithm is well defined, we will show that the nonempty set $K^{*}$ is always contained in $H_{k}^{1}\cap H_{k}^{2} \cap X$ for the projection step.

### Lemma 3.2

*Let Assumption *2.1 *be true*. *Then we have* $K^{*}\subseteq H_{k}^{1}\cap H_{k}^{2} \cap X$ *for all* $k\geq0$.

### Proof

From the analysis in Lemma 3.1, it is sufficient to prove that $K^{*}\subseteq H_{k}^{2}$ for all $k\geq0$. By induction, if $k=0$, it is obvious that

$$K^{*}\subseteq H_{0}^{2}= \mathbb{R}^{n}. $$

Suppose that

$$K^{*}\subseteq H_{k}^{2} $$

holds for $k=l\geq0$. Then

$$K^{*}\subseteq H_{l}^{1}\cap H_{l}^{2} \cap X. $$

For any $x^{*}\in K^{*}$, by Lemma 2.1 and the fact that

$$x^{l+1}=P_{H_{l}^{1}\cap H_{l}^{2} \cap X}\bigl(x^{0}\bigr), $$

we know that

$$\bigl\langle x^{*}-x^{l+1}, x^{0}-x^{l+1} \bigr\rangle \leq0. $$

Thus $K^{*}\subseteq H_{l+1}^{2}$, which means that $K^{*}\subseteq H_{k}^{2}$ for all $k\geq0$ and the desired result follows. □

In the following, we show the expansion property of the algorithm with respect to the initial point.

### Lemma 3.3

*Suppose* $\{x^{k}\}$ *is the generated sequence of Algorithm *3.1, *we have*

$$\bigl\Vert x^{k+1}-x^{k} \bigr\Vert ^{2}+ \bigl\Vert x^{k}-x^{0} \bigr\Vert ^{2}\leq \bigl\Vert x^{k+1}-x^{0} \bigr\Vert ^{2}. $$

### Proof

By Algorithm 3.1, one has

$$x^{k+1}=P_{H_{k}^{1}\cap H_{k}^{2} \cap X}\bigl(x^{0}\bigr). $$

So $x^{k+1} \in H_{k}^{2}$ and

$$P_{H_{k}^{2}}\bigl(x^{k+1}\bigr)=x^{k+1}. $$

By the definition of $H_{k}^{2}$, we have

$$\bigl\langle z-x^{k}, x^{0}-x^{k} \bigr\rangle \leq0, \quad \forall z\in H_{k}^{2}. $$

Thus, $x^{k}=P_{H_{k}^{2}}(x^{0})$ from the Remark 2.1. Then, from Lemma 2.1, we obtain

$$\bigl\Vert P_{H_{k}^{2}}\bigl(x^{k+1}\bigr)-P_{H_{k}^{2}} \bigl(x^{0}\bigr) \bigr\Vert \leq \bigl\Vert x^{k+1}-x^{0} \bigr\Vert ^{2}- \bigl\Vert P_{H_{k}^{2}}\bigl(x^{k+1} \bigr)-x^{k+1}+x^{0}-P_{H_{k}^{2}}\bigl(x^{0} \bigr) \bigr\Vert ^{2}, $$

which can be written as

$$\bigl\Vert x^{k+1}-x^{k} \bigr\Vert ^{2}\leq \bigl\Vert x^{k+1}-x^{0} \bigr\Vert ^{2}- \bigl\Vert x^{k}-x^{0} \bigr\Vert ^{2}, $$

*i.e.*,

$$\bigl\Vert x^{k+1}-x^{k} \bigr\Vert ^{2}+ \bigl\Vert x^{k}-x^{0} \bigr\Vert ^{2}\leq \bigl\Vert x^{k+1}-x^{0} \bigr\Vert ^{2}, $$

and the proof is completed. □

To prove the boundedness of the generated sequence $\{x^{k}\}$, we assume that the algorithm generates an infinite sequence for simple.

### Lemma 3.4

*Suppose Assumption *2.1 *is true*. *Then the generated sequence* $\{x^{k}\}$ *of Algorithm *3.1 *is bounded*.

### Proof

By Assumption 2.1, we know that $K^{*}\neq \emptyset$. Since $x^{k+1}$ is the projection of $x^{0}$ onto $H_{k}^{1}\cap H_{k}^{2} \cap X$, by Lemma 3.2 and the definition of projection, we have

$$\bigl\Vert x^{k+1}-x^{0} \bigr\Vert \leq \bigl\Vert x^{*}-x^{0} \bigr\Vert , \quad\forall x^{*}\in K^{*}. $$

So, $\{x^{k}\}$ is a bounded sequence. □

Since $\{x^{k}\}$ is bounded, it has an accumulation point. Without loss of generality, assume that the subsequence $\{x^{k_{j}}\}$ converges to *x̄*. Then the sequences $\{y^{k_{j}}\}$, $\{z^{k_{j}}\}$ and $\{g^{k_{j}}\}$ are bounded from the Proposition 10 in [23], where $g^{k_{j}} \in\partial f(z^{k_{j}},x^{k_{j}})$.

Before given the next result, the following lemma is needed (for details see [23]).

### Lemma 3.5

*For every* $y\in K(x^{k})$, *we have*

$$f\bigl(x^{k},y\bigr)\geq f\bigl(x^{k},y^{k}\bigr)+ \bigl\langle x^{k}-y^{k}, y-y^{k}\bigr\rangle . $$

*In particular*, $f(x^{k},y^{k})+ \Vert x^{k}-y^{k} \Vert^{2}\leq0$.

### Lemma 3.6

*Suppose* $\{x^{k_{j}}\}$ *is the sequence presented as in Lemma *3.4. *If* $x^{k_{j}}\neq y^{k_{j}}$, *then*

$$\bigl\Vert x^{k_{j}}-y^{k_{j}} \bigr\Vert \rightarrow0 $$

*as* $j\rightarrow\infty$.

### Proof

We distinguish for the proof two cases.

(1) If $\liminf_{k\rightarrow\infty}\eta_{k}>0$, by Lemma 3.4, one has

$$\bigl\Vert x^{k+1}-x^{k} \bigr\Vert ^{2}+ \bigl\Vert x^{k}-x^{0} \bigr\Vert ^{2}\leq \bigl\Vert x^{k+1}-x^{0} \bigr\Vert ^{2}. $$

Thus, the sequence $\{ \Vert x^{k}-x^{0} \Vert\}$ is nondecreasing and bounded, and hence convergent, which implies that

$$\lim_{k\rightarrow\infty} \bigl\Vert x^{k+1}-x^{k} \bigr\Vert =0. $$

On the other hand, by Assumption 2.1(i) and the fact that

$$z^{k_{j}}=(1-\eta_{k_{j}})x^{k_{j}}+\eta_{k_{j}} y^{k_{j}}, $$

we have

$$0=f\bigl(z^{k_{j}},z^{k_{j}}\bigr)\leq(1-\eta_{k_{j}})f \bigl(z^{k_{j}}, x^{k_{j}}\bigr)+ \eta _{k_{j}} f \bigl(z^{k_{j}},y^{k_{j}}\bigr), $$

which can be written as

$$-f\bigl(z^{k_{j}},y^{k_{j}}\bigr)\leq\biggl(\frac{1}{\eta_{k_{j}}}-1 \biggr)f\bigl(z^{k_{j}},x^{k_{j}}\bigr). $$

By (3.1), one has

$$f\bigl(z^{k_{j}},x^{k_{j}}\bigr)\geq c\eta_{k_{j}} \bigl\Vert x^{k_{j}}-y^{k_{j}} \bigr\Vert ^{2}>0. $$

Then we will prove that

3.2 $$ P_{H_{k_{j}}^{1}}\bigl(x^{k_{j}}\bigr)=x^{k_{j}}- \frac{f(z^{k_{j}},x^{k_{j}})}{ \Vert g^{k_{j}} \Vert ^{2}}g^{k_{j}}, $$

where $g^{k_{j}} \in\partial f(z^{k_{j}},x^{k_{j}})$. For all $x \in H_{k_{j}}^{1}$, from the remark of Lemma 2.1, we only need to prove

$$\biggl\langle x^{k_{j}}-\biggl(x^{k_{j}}-\frac{f(z^{k_{j}},x^{k_{j}})}{ \Vert g^{k_{j}} \Vert ^{2}}g^{k_{j}} \biggr), \biggl(x^{k_{j}}-\frac{f(z^{k_{j}},x^{k_{j}})}{ \Vert g^{k_{j}} \Vert ^{2}}g^{k_{j}}\biggr)-x\biggr\rangle \geq0, $$

*i.e.*,

$$\frac{f(z^{k_{j}},x^{k_{j}})}{ \Vert g^{k_{j}} \Vert ^{2}}\bigl\langle g^{k_{j}}, x^{k_{j}}-x \bigr\rangle - \frac{f^{2}(z^{k_{j}},x^{k_{j}})}{ \Vert g^{k_{j}} \Vert ^{2}}\geq0, $$

which is equivalent to

3.3 $$ \bigl\langle g^{k_{j}}, x-x^{k_{j}} \bigr\rangle +f \bigl(z^{k_{j}},x^{k_{j}}\bigr)\leq0. $$

Since $g^{k_{j}} \in\partial f(z^{k_{j}},x^{k_{j}})$, by the definition of subdifferential we have

$$f\bigl(z^{k_{j}},x\bigr)\geq f\bigl(z^{k_{j}},x^{k_{j}} \bigr)+\bigl\langle g^{k_{j}}, x-x^{k_{j}} \bigr\rangle , \quad \forall x\in\mathbb{R}^{n}. $$

So, from the definition of $H_{k_{j}}^{1}$, for all $x\in H_{k_{j}}^{1}$ we have

$$f\bigl(z^{k_{j}},x\bigr)\leq0, $$

which implies that (3.3) holds. Moreover, (3.2) is right.

By (3.2) and the fact that there is a constant $M>0$ such that $\Vert g^{k_{j}} \Vert\leq M$, we obtain

$$\bigl\Vert x^{k_{j}}-x^{{k_{j}}+1} \bigr\Vert \geq \bigl\Vert x^{k_{j}}-P_{H_{k_{j}}^{1}}\bigl(x^{k_{j}}\bigr) \bigr\Vert = \frac {f(z^{k_{j}},x^{k_{j}})}{ \Vert g^{k_{j}} \Vert }\geq\frac {c\eta_{k_{j}}}{M} \bigl\Vert x^{k_{j}}-y^{k_{j}} \bigr\Vert ^{2}, $$

which implies that $\Vert x^{k_{j}}-y^{k_{j}} \Vert\rightarrow0$, $j\rightarrow\infty$, and the desired result holds.

(2) Suppose that $\liminf_{k\rightarrow\infty}\eta_{k}=0$, and for any subsequence $\{\eta_{k_{j}}\}$ of $\{\eta_{k}\}$, it satisfies

$$\lim_{j\rightarrow\infty}\eta_{k_{j}}=0. $$

Let $\{x^{k_{j}}\}\rightarrow\bar{x}$ as $j\rightarrow\infty$, it follows that

$$\bar{z}^{k_{j}}=\biggl(1-\frac{\eta_{k_{j}}}{\gamma}\biggr)x^{k_{j}}+ \frac{\eta _{k_{j}}}{\gamma}y^{k_{j}}\rightarrow\bar{x}, \quad j\rightarrow\infty. $$

By the definition of $\{\eta_{k_{j}}\}$, one has

$$f\bigl(\bar{z}^{k_{j}},x^{k_{j}}\bigr)-f\bigl( \bar{z}^{k_{j}},y^{k_{j}}\bigr)< c \bigl\Vert x^{k_{j}}-y^{k_{j}} \bigr\Vert ^{2}. $$

Let *ȳ* be the limit of $\{y^{k_{j}}\}$. By Lemma 3.5 we have

$$f\bigl(\bar{z}^{k_{j}},x^{k_{j}}\bigr)-f\bigl( \bar{z}^{k_{j}},y^{k_{j}}\bigr)< c \bigl\Vert x^{k_{j}}-y^{k_{j}} \bigr\Vert ^{2}\leq-cf\bigl(x^{k_{j}},y^{k_{j}}\bigr). $$

Taking $j\rightarrow\infty$ and remembering the fact that *f* is continuous, we obtain

$$f(\bar{x},\bar{x})-f(\bar{x},\bar{y})\leq-cf(\bar{x},\bar{y}), $$

which implies that $f(\bar{x},\bar{y})\geq0$. So $\Vert x^{k_{j}}-y^{k_{j}} \Vert\rightarrow0$, $j\rightarrow\infty$ and the desired result follows. □

Based on the analysis above, we can establish the main results of this section that the generated sequence $\{x^{k}\}$ globally converge to a solution of the problem (1.1).

### Theorem 3.1

*Suppose* $\{x^{k}\}$ *is an infinite sequence generated in Algorithm *3.1. *Let conditions of Lemma *3.6 *be true*. *Then each accumulation point of* $\{x^{k}\}$ *is a solution of the QEP under the Assumption *2.1.

### Proof

By Lemma 3.4, without loss of generality, assume that the subsequence $\{x^{k_{j}}\}$ converges to *x̄*. By Lemma 3.6, one has $\Vert x^{k_{j}}-y^{k_{j}} \Vert \rightarrow0$ and

$$y^{k_{j}}=y^{k_{j}}-x^{k_{j}} +x^{k_{j}}\to\bar{x}, $$

where $y^{k_{j}} \in K(x^{k_{j}})$ for every *j*. Thus $\bar{x} \in K(\bar {x})$ from the fact that *K* is upper semicontinuous.

To prove that *x̄* is a solution of the problem (1.1), since

$$y^{k}=\arg\min_{y\in K(x^{k})} \biggl[f\bigl(x^{k},y \bigr)+\frac{1}{2} \bigl\Vert y-x^{k} \bigr\Vert ^{2} \biggr], $$

the optimality condition implies that there exists $\omega\in\partial f(x^{k},y^{k})$ such that

$$0=\omega+y^{k}-x^{k}+s^{k}, $$

where $s^{k} \in N_{K(x^{k})}(y^{k})$ is a vector in the normal cone to $K(x^{k})$ at $y^{k}$. Then we have

3.4 $$ \bigl\langle y^{k}-x^{k}, y-y^{k} \bigr\rangle \geq\bigl\langle \omega, y^{k}-y \bigr\rangle , \quad\forall y \in K\bigl(x^{k}\bigr). $$

On the other hand, since $\omega\in\partial f(x^{k},y^{k})$ and by the well-known Moreau–Rockafellar theorem [35], one has

3.5 $$ f\bigl(x^{k},y\bigr)-f\bigl(x^{k},y^{k} \bigr) \geq\bigl\langle \omega, y-y^{k}\bigr\rangle . $$

By (3.4) and (3.5), we have

3.6 $$ f\bigl(x^{k},y\bigr)-f\bigl(x^{k},y^{k} \bigr) \geq\bigl\langle x^{k}-y^{k}, y-y^{k}\bigr\rangle , \quad\forall y\in K\bigl(x^{k}\bigr). $$

Letting $k=k_{j}$ in (3.6)

$$f\bigl(x^{k_{j}},y\bigr)-f\bigl(x^{k_{j}},y^{k_{j}}\bigr) \geq\bigl\langle x^{k_{j}}-y^{k_{j}}, y-y^{k_{j}}\bigr\rangle , \quad\forall y\in K\bigl(x^{k_{j}}\bigr). $$

Taking $j\rightarrow\infty$ and remembering that *f* is continuous, we obtain

$$f(\bar{x}, y) \geq0, \quad\forall y \in K(\bar{x}), $$

that is, *x̄* is a solution of the QEP and the proof is completed. □

### Theorem 3.2

*Under the assumption of Theorem *3.1, *the generated sequence* $\{x^{k}\}$ *converges to a solution* $x^{*}$ *such that*

$$x^{*}=P_{K^{*}}\bigl(x^{0}\bigr) $$

*under the Assumption *2.1.

### Proof

By Theorem 3.1, we know that the sequence $\{x^{k}\}$ is bounded and that every accumulation point $x^{*}$ of $\{x^{k}\}$ is a solution of the problem (1.1). Let $\{x^{k_{j}}\}$ be a convergent subsequence of $\{x^{k}\}$, and let $x^{*}\in K^{*}$ be its limit. Let $\bar{x}=P_{K^{*}}(x^{0})$. Then by Lemma 3.2,

$$\bar{x} \in H_{k_{j}-1}^{1}\cap H_{k_{j}-1}^{2} \cap X, $$

for all *j*. So, from the iterative procedure of Algorithm 3.1,

$$x^{k_{j}}=P_{H_{k_{j}-1}^{1}\cap H_{k_{j}-1}^{2} \cap X}\bigl(x^{0}\bigr), $$

one has

3.7 $$ \bigl\Vert x^{k_{j}}-x^{0} \bigr\Vert \leq \bigl\Vert \bar{x}-x^{0} \bigr\Vert . $$

Thus,

$$\begin{aligned} \bigl\Vert x^{k_{j}}-\bar{x} \bigr\Vert ^{2}&= \bigl\Vert x^{k_{j}}-x^{0}+x^{0}- \bar{x} \bigr\Vert ^{2} \\ &= \bigl\Vert x^{k_{j}}-x^{0} \bigr\Vert ^{2}+ \bigl\Vert x^{0}-\bar{x} \bigr\Vert ^{2}+2\bigl\langle x^{k_{j}}-x^{0}, x^{0}-\bar{x}\bigr\rangle \\ &\leq \bigl\Vert \bar{x}-x^{0} \bigr\Vert ^{2}+ \bigl\Vert x^{0}-\bar {x} \bigr\Vert ^{2}+2\bigl\langle x^{k_{j}}-x^{0}, x^{0}-\bar{x}\bigr\rangle , \end{aligned} $$

where the inequality follows from (3.7). Letting $j\rightarrow\infty$, it follows that

3.8 $$ \begin{aligned}[b]\limsup_{j\rightarrow\infty} \bigl\Vert x^{k_{j}}-\bar {x} \bigr\Vert ^{2}&\leq 2 \bigl\Vert \bar{x}-x^{0} \bigr\Vert ^{2}+2\bigl\langle x^{*}-x^{0}, x^{0}-\bar{x}\bigr\rangle \\ &= 2\bigl\langle x^{*}-\bar{x}, x^{0}-\bar{x}\bigr\rangle . \end{aligned} $$

Due to Lemma 2.1 and the fact that $\bar{x}=P_{K^{*}}(x^{0})$ and $x^{*} \in K^{*}$, we have

$$\bigl\langle x^{*}-\bar{x}, x^{0}-\bar{x}\bigr\rangle \leq0. $$

Combining this with (3.8) and the fact that $x^{*}$ is the limit of $\{x^{k_{j}}\}$, we conclude that the sequence $\{x^{k_{j}}\}$ converges to *x̄* and

$$x^{*}=\bar{x}=P_{K^{*}}\bigl(x^{0}\bigr). $$

Since $x^{*}$ was taken as an arbitrary accumulation point of $\{x^{k}\}$, it follows that *x̄* is the unique accumulation point of this sequence. Since $\{ x^{k}\}$ is bounded, the whole sequence $\{x^{k}\}$ converges to *x̄*. □

## Numerical experiment

In this section, we will make some numerical experiments and give a numerical comparison with the method proposed in [23] to test the efficiency of the proposed method. The MATLAB codes are run on a PIV 2.0 GHz personal computer under MATLAB version 7.0.1.24704(R14). In the following, ‘Iter.’ denotes the number of iteration, and ‘CPU’ denotes the running time in seconds. The tolerance *ε* means the iterative procedure terminates when $\Vert x^{k}-y^{k} \Vert\leq\varepsilon$.

### Example 4.1

The bifunction *f* of the quasi-equilibrium problem is defined for each $x, y \in R^{5}$ by

$$f(x,y)=\langle Px+Qy+q, y-x\rangle, $$

where *q*, *P*, *Q* are chosen as follows:

$$q=\left [ \begin{matrix} 1 \\ -2 \\ -1 \\ 2 \\ -1 \end{matrix} \right ];\qquad P=\left [ \begin{matrix} 3.1 & 2 & 0 & 0 & 0\\ 2 & 3.6 & 0 & 0 & 0\\ 0 & 0 & 3.5 & 2 & 0\\ 0 & 0 & 2 & 3.3 & 0\\ 0 & 0 & 0 & 0 & 3 \end{matrix} \right ];\qquad Q=\left [ \begin{matrix} 1.6 & 1 & 0 & 0 & 0\\ 1 & 1.6 & 0 & 0 & 0\\ 0 & 0 & 1.5 & 1 & 0\\ 0 & 0 & 1 & 1.5 & 0\\ 0 & 0 & 0 & 0 & 2 \end{matrix} \right ]. $$

The moving set $K(x)=\prod_{1\leq i\leq5}K_{i}(x)$ where for each $x\in R^{5}$ and each *i*, the set $K_{i}(x)$ is defined by

$$K_{i}(x)=\biggl\{ y_{i}\in R \mid y_{i} + \sum _{1\leq j\leq5, j\neq i}x_{j}\geq 1\biggr\} . $$

This problem was tested in [36] with initial point $x^{0}=(1, 3, 1, 1, 2)^{T}$. They obtained the appropriate solution after 21 iterates with the tolerance $\varepsilon=10^{-3}$.

By the algorithm proposed in this paper, the numerical results obtained for this example are listed in Table 1 with $c=\gamma=0.5$, $\varepsilon=10^{-3}$ and $X=K(x)$, and with different initial points.

**Table 1:** Numerical results for Example 4.1

	Initial point $x^{0}$				
	$(0, 0, 0, 0, 0)^{T}$	$(1, 3, 1, 1, 2)^{T}$	$(1, 1, 1, 1, 2)^{T}$	$(1, 0, 1, 0, 2)^{T}$	$(0, 1, 1, 0, 2)^{T}$
Iter.	5	13	9	7	8
CPU	0.2060	0.5340	0.3590	0.2190	0.3430

Now, we consider a quasi-variational inequality problems and we solve it by using Algorithm 3.1 with the equilibrium function $f(x, y)= \langle F(x), y-x\rangle$.

### Example 4.2

Consider a two-person game whose QVI formulation involves the function $F=(F_{1}, F_{2})$ and the multi-valued mapping $K(x)=K_{1}(x_{2})\times K_{2}(x_{1})$ for each $x=(x_{1}, x_{2})\in R^{2}$, where

$$F_{1}(x)=2x_{1}+\frac{8}{3}x_{2}-34,\qquad F_{2}(x)=2x_{2}+\frac{5}{4}x_{1}-24.25, $$

and

$$\begin{aligned}& K_{1}(x_{2})=\{y_{1}\in R \mid 0\leq y_{1} \leq10, y_{1}\leq15-x_{2}\}, \\& K_{2}(x_{1})=\{y_{2}\in R \mid 0\leq y_{2} \leq10, y_{2}\leq15-x_{1}\}. \end{aligned}$$

This problem was tested in [23]. The numerical results of Algorithm 3.1, abbreviated as Alg. 31, for this example are shown in Table 2 with different initial points.

**Table 2:** Numerical results for Example 4.2

Alg.31	Initial point $x^{0}$					
	(10,10)	(10,0)	(9,1)	(9,3)	(9,9)	(8,10)
Iter.	53	2	2	8	51	48
CPU(s)	0.7030	0.0160	0.0310	0.2030	0.8440	0.7810

For this example, we choose $X=K(x)$ and take $c=\gamma=0.5$. During the experiments, we set the stopping criterion $\varepsilon =10^{-6}$. The numerical comparison of our proposed method with the algorithms, *i.e.*, Alg.1, Alg.1a, Alg.1b, proposed in [23] are given in Tables 3 and 4.

**Table 3:** Iterations from Alg.31, Alg.1, Alg.1a and Alg.1b respectively

Initial point	Number of iterations			
	Alg.31	Alg.1	Alg.1a	Alg.1b
(10,0)	2	3	2	2
(10,10)	53	255	120	120

**Table 4:** The CPU time from Alg.31, Alg.1, Alg.1a and Alg.1b respectively

Initial point	CPU (s)			
	Alg.31	Alg.1	Alg.1a	Alg.1b
(10,0)	0.02	0.26	0.20	0.15
(10,10)	0.70	8.43	3.70	2.57

## Conclusions

In this paper, we have proposed a new type of extragradient projection method for the quasi-equilibrium problem. The generated sequence by the newly designed method possesses an expansion property with respect to the initial point. The existence results of the problem is established under pseudomonotonicity condition of the equilibrium function and the continuity of the underlying multi-valued mapping. Furthermore, we have shown that the generated sequence converges to the nearest point in the solution set to the initial point. The given numerical experiments show the efficiency of the proposed method.
